# Multidisciplinary En-Bloc Resection of Sacral Chordoma: A Narrative Review and Illustrative Case

**DOI:** 10.3390/jcm14134480

**Published:** 2025-06-24

**Authors:** Daniel Kiss-Bodolay, Frederic Ris, Adrien Lavalley, Aria Nouri, Carlo M. Oranges, Guillaume Meurette, Karl Schaller, Enrico Tessitore, Granit Molliqaj

**Affiliations:** 1 Faculty of Medicine, University of Geneva, 1205 Geneva, Switzerland; frederic.ris@hug.ch (F.R.); adrien.lavalley@hug.ch (A.L.); aria.nouri@hug.ch (A.N.); karl.schaller@hug.ch (K.S.); enrico.tessitore@hug.ch (E.T.); granit.molliqaj@hug.ch (G.M.); 2 Division of Neurosurgery, Department of Clinical Neurosciences, University Hospitals of Geneva, HUG, 1205 Geneva, Switzerland; 3 Division of Visceral Surgery, Department of Surgery, University Hospitals of Geneva, HUG, 1205 Geneva, Switzerland; guillaume.meurette@hug.ch; 4 Division of Plastic Surgery, Department of Surgery, University Hospitals of Geneva, HUG, 1205 Geneva, Switzerland; carlo.oranges@hug.ch

**Keywords:** sacral chordoma, sacrectomy, en-bloc resection, multidisciplinary management, sacral anatomy

## Abstract

**Background/Objectives**: Sacral chordomas are rare, locally invasive tumors that pose significant surgical and oncological challenges due to their anatomical complexity, proximity to critical structures, and resistance to conventional therapies. **Methods**: A literature search focused on contemporary multidisciplinary management of sacral chordoma was conducted. An illustrative case of such a multidisciplinary approach is presented. **Results**: Achieving optimal outcomes necessitates a multidisciplinary approach that balances en-bloc resection with negative margins and preservation of biomechanical stability and neurological function. Negative resection margins are a key determinant of long-term survival and reduced recurrence, particularly for tumors involving the upper sacrum (S1–S2). While postoperative radiation therapy provides adjunctive benefits, precision in surgical planning and execution remains paramount. Emerging technologies, such as augmented reality and 3D-printed anatomical models, are enhancing surgical precision, while the role of multidisciplinary surgical teams in improving outcomes requires further study. **Conclusions**: This review highlights the complexities of sacral chordoma management, focusing on surgical strategies, functional trade-offs, and future directions to optimize oncological and functional outcomes.

## 1. Introduction

Sacral chordoma is a rare malignant tumor arising from embryonic notochordal remnants. Although slow-growing and indolent, it is locally invasive and presents significant surgical challenges upon diagnosis [1]. Optimal disease-free survival is closely linked to a multidisciplinary treatment approach, typically involving en-bloc resection and proton beam radiation therapy [1,2,3,4,5,6,7]. While minimizing treatment-related morbidity is crucial, achieving an optimal extent of resection requires a tailored degree of invasiveness, including variable extensions of sacrectomy, sacral nerve root sacrifice, pelvic exenteration, and complex tissue reconstruction for wound closure [1,8,9].

Evidence strongly supports the preservation of sacral roots, as ambulatory function, bowel, bladder, and anal sphincter functions depend on them [10,11,12,13,14,15]. The use of 3D reconstruction techniques based on multimodal imaging, alongside advancements in intraoperative navigation tools, has significantly improved the safety of sacral osteotomies, dissection around sacral nerve roots, and screw fixation in cases of spino-pelvic instability [16,17,18,19,20,21,22,23,24]. Additionally, neuromonitoring is routinely employed to minimize nerve root injury [25].

Optimized outcomes are facilitated by the involvement of a multidisciplinary surgical team, which typically includes visceral surgeons, plastic surgeons, and neurosurgeons with expertise in oncological spine treatment. Sacral chordomas treated by a multidisciplinary team are generally larger, more infiltrative in the sacrum, and extend closer to the skin [26]. However, these complex cases often result in a higher risk of complications [1,23,26]. Complications are frequently associated with factors such as tumor size, sacral extension, involvement of critical structures like the iliac vessels, and the technical challenges of using an anterior surgical approach [26].

In our department, this intervention is performed jointly by the visceral surgery and plastic surgery teams in a three-stage manner under 3D navigation guidance and neuromonitoring. In this work, we present a comprehensive review of the literature on the surgical treatment modalities of sacral chordomas, along with an illustrative case demonstrating multidisciplinary surgical management to achieve en-bloc resection of a large sacral chordoma.

## 2. Materials and Methods

The literature search was conducted in MEDLINE, Google Scholar and PubMed. Relevant studies about the surgical and multidisciplinary management of sacral chordoma were identified by screening the titles and abstracts. Clinical and imaging data, and intraoperative pictures were collected from a case of multidisciplinary surgical management of a rapidly growing sacral chordoma. Preoperative 3D anatomical reconstructions were generated using cranial planning software (Elements Brainlab AG, Feldkirchen, Germany).

## 3. Results: Narrative Review

### 3.1. Multidisciplinary Surgical Management

Achieving optimal resection margins is a crucial prognostic factor in sacral chordoma management due to the poor sensitivity of these tumors to chemotherapy and radiation, as well as their high propensity for recurrence [27,28,29,30,31,32]. Research consistently shows that en-bloc resection without penetrating the tumor’s capsule correlates with reduced recurrence, but its association with prolonged survival remains unclear [9,33,34,35,36,37,38,39,40,41]. Although promising results have been observed with the combination of en-bloc resection and proton radiation therapy, the overall long-term outcomes of resected sacral chordomas remain poor [5,7,42]. Fewer than 50% of patients treated with R0 resection show no evidence of recurrence at 15 years [3,43,44,45]. In a large series of 101 cases, overall survival at 5 and 10 years for primary tumors was 79% and 59%, respectively, while for recurrent cases, overall survival was 65% and 40% at 5 and 10 years, respectively [46].

Early diagnosis remains a significant challenge, as chordomas exhibit indolent and invasive growth patterns. By the time of diagnosis, most patients present with extra-compartmental extension [8,34,47]. Large tumor volumes and high sacral tumors (above S2) are associated with unfavorable surgical outcomes [39,48,49,50,51]. Undiagnosed chordomas frequently invade the sacral foramina and the neural canal, extend to the sacroiliac joints and ilium, and infiltrate muscles and soft tissues, including the skin around the pelvis [28]. Worse overall survival and higher local recurrence rates are correlated with infiltration of subcutaneous fat, sacroiliac joints, and sacral muscle compartments [52,53]. Additionally, erosion of ligamentous anatomy leads to biomechanical instability [54]. Despite the presence of the presacral fascia separating the pelvic compartment, rectal involvement through anterior extension is not uncommon [55,56,57].

Surgical resection of large sacral tumors presents a technical challenge due to the complex vascular and nervous anatomy, often necessitating multistage approaches. In specialized oncological centers, patients with larger and more invasive tumors undergo multidisciplinary surgical management aimed at reducing perioperative risks associated with the anatomical and oncological complexity of sacral chordomas [1,9,23,26,58]. Multidisciplinary management typically involves neurosurgeons specialized in spine surgery and general or colorectal surgeons performing abdominopelvic organ mobilization and vessel control to achieve optimal resection margins [1,4,9,24,58,59]. Furthermore, plastic and reconstructive surgery plays a key role in mitigating wound-related complications, particularly in cases of en-bloc resection with large tissue defects, by employing reconstructive strategies [60,61,62,63,64,65].

It remains uncertain whether multidisciplinary management, longer surgical times, and the involvement of additional surgeons directly lead to improved patient outcomes [1,26,66,67,68,69]. While these approaches may address complex cases more comprehensively, they also introduce risks such as increased perioperative complications, underscoring the need for further research to clarify their impact. Notably, hospitalization duration is significantly longer, and postoperative bladder function outcomes are worse in patients treated for complex tumors, as reported by Schilling et al. [26].

### 3.2. Surgical Technique

There is still an ongoing debate about the optimal approach for en-bloc resection of the tumor between a combined anterior/posterior and a posterior-only approach. The choice of surgical approach is primarily determined by the tumor’s location. Smaller chordomas located caudally to the third sacral vertebra (S3) that do not involve the rectum or extend significantly into the pelvis are often manageable with a posterior-only approach, offering sufficient access while reducing the need for more invasive procedures [1,8,14,48,64]. In these cases, the patient is placed in a prone position, and the tumor is resected en-bloc with minimal soft-tissue disruption, typically sparing the anorectum.

For tumors extending cephalad to S3, a combined anterior-posterior approach is often preferred to provide safer access to critical structures, including viscera and major vessels [1,8,14,48,64]. The combined anterior-posterior approach is also necessary for larger tumors extending anteriorly or when the rectum and pelvic floor muscles are involved [1,8,14,48,64]. The anterior stage allows for better visualization of tumor borders, and when necessary, rectal amputation is performed en-bloc with the tumor [1,8,14,48,64]. To complete this approach, an omental flap may be placed within the pelvis, and a colostomy may be created if bowel function preservation is not possible.

Advanced imaging techniques have revolutionized the planning and precision of chordoma resection. Preoperative MRI and CT scans provide detailed maps of tumor invasion into soft tissues and bones, while intraoperative navigation assists surgeons in executing precise osteotomies [1,24,70]. Augmented reality and 3D-printed anatomical models represent emerging technologies that offer real-time assistance in tumor localization and planning, potentially enabling more conservative approaches when applicable [1,24,71]. Although the evidence supporting the optimal approach remains mixed, studies indicate that the combined approach may allow for better control of resection margins in complex or more cephalad tumors [1,8,14,48,64]. Nevertheless, R0 tumor resection was reported to be superior in a combined anteroposterior approach with an inferior rate of wound-related complication and reoperation rate [72].

### 3.3. Trade-Off Between Oncological Goal and Neurological Function

Different levels of margin control and nerve preservation significantly impact both oncological outcomes and patient quality of life. The goal of surgery is to achieve an en-bloc resection with safe margins while preserving as much function as possible. However, many patients develop urinary sphincter issues, sexual dysfunction, and sometimes weakness of the lower limbs due to the proximity of the sacral nerve roots [28].

The sacrum serves as the caudal crossroad of many critical vasculo-nervous structures, such as the lumbosacral trunk and sacral plexus, which are essential for ambulatory capabilities (e.g., the sciatic nerve, L4–S3) as well as sexual, bowel, and bladder functions (e.g., the pudendal nerve, S2–S4, which innervates the external anal canal) [73]. When tumors are located close to the dura or significant nerve structures, surgical decisions often involve a trade-off between achieving oncological goals and preserving neurological function. The level of sacrectomy closely correlates with the expected severity of postoperative neurological deficits [38].

In cases where the S1 root is sacrificed, patients typically experience significant motor impairment, sphincter dysfunction, and sexual dysfunction [74]. When both S2 roots are preserved, at least half of patients maintain ambulatory capabilities, but saddle anesthesia and sphincter dysfunction are not uncommon [10,11,15]. Preserving at least one S2 root has been associated with better urinary and fecal continence outcomes [75,76]. Preserving S3 roots correlates with better bladder and bowel function, although sexual dysfunction may still occur [10,11,15,36,74,77].

Overall, these findings highlight the critical difference between middle sacral resections (e.g., S3) and high sacral resections (e.g., S1/S2), the latter being associated with severe functional loss [4,36]. In cases of nerve injury or partial nerve sacrifice, microsurgical reconstruction using sural nerve grafts has been reported to be effective in restoring sexual and sphincter functions [78].

### 3.4. Sacrectomy and Mechanical Stability

The complex pelvic anatomy often limits the ability to achieve wide margins in 35–75% of cases, particularly in proximal sacral tumors at the S1 or S2 levels. These tumors frequently infiltrate the sacroiliac joints (SIJs) and adjacent musculature, jeopardizing biomechanical stability [39,46,79,80,81,82,83,84]. Tumor invasion into the SIJs or musculature significantly increases recurrence risks due to potential micro-satellite lesions that often remain undetected.

A study by Stener and Gutenberg demonstrated that resecting one-third of the SIJ weakens the pelvic ring by approximately 30%, while resection of tissue between the first and second sacral vertebrae reduces stability by as much as 50% [75]. Additional cadaveric biomechanical studies confirmed that transverse partial sacrectomy at or above the S1/S2 level can lead to both rotational and compressive instability [85,86]. Furthermore, a recent clinical study observed a higher risk of postoperative sacral fractures following en-bloc chordoma resection involving S1/S2 amputation, partial SIJ resection, and a combined anteroposterior surgical approach [87]. In contrast, significant changes in pelvic incidence were noted with sacrectomy but not with subtotal sacral amputation, including partial SIJ resection [88].

This mechanical compromise highlights the necessity of internal fixation to restore spinopelvic stability, particularly in trans-S1 resections [85,86,89]. Total sacrectomy, which results in complete spinopelvic dissociation, requires extensive stabilization procedures to reestablish structural support and allow ambulation. However, these procedures are associated with a significant mechanical fixation failure rate of approximately 25% [90].

Several reconstruction techniques are available to address sacroiliac postoperative instability. Common methods include double iliac screw fixation and the Galveston technique [12,13,51]. Lumbopelvic reconstruction techniques are continually being refined, and recently, novel patient-specific sacral implants have been developed for biomechanical reconstruction following total sacrectomy [91,92,93].

### 3.5. Reconstruction Strategies for Sacral Defects

It has been reported that between one-third and two-thirds of patients experience wound complications after sacrectomy, such as postoperative surgical site infection or dehiscence, likely due to tissue defects and the effects of adjuvant radiotherapy [94,95,96]. Still under debate, factors such as prior radiation, rectal rupture, younger age (<40 years), diabetes mellitus, tumor diameter (≥10 cm), and instrumentation have been suggested as potential contributors to an increased risk of wound complications [94,95,96].

Another potential complication after sacrectomy is sacral herniation of abdominal contents, which can result from atrophy of denervated pelvic floor muscles due to the loss of S2–S5 innervation [1,97]. As such, ensuring proper wound healing and soft-tissue reconstruction with well-vascularized flaps is critical. In multidisciplinary management, plastic and reconstructive surgeons employ various techniques to repair large tissue defects following total or partial sacrectomy for chordomas. Common approaches include local gluteal myocutaneous flap advancement, rotational posterior thigh flaps, and vertical rectus abdominis myocutaneous (VRAM) flaps, among others [60,62,65,98,99,100,101,102,103,104,105,106].

Prior studies have evaluated sacrectomy wound outcomes managed by multidisciplinary teams and recommend VRAM flaps as the gold standard reconstruction technique post-sacrectomy due to their low infection rates and superior ability to obliterate dead space compared to local gluteal flaps [63,99,100,107]. However, even with VRAM flaps, complication rates remain significant. Houdek et al. reported wound complications in 47% of 87 patients undergoing VRAM flap reconstruction for post-sacrectomy defects, with 51% requiring reoperation, and 57% of these reoperations were due to wound dehiscence or infection [60].

In general, VRAM flaps are preferred for larger defects, irradiated regions, and cases with compromised gluteal vasculature, such as when the internal iliac artery has been sacrificed. Conversely, local gluteal musculocutaneous flaps, omental flaps, and the recently described double-pedicled gracilis muscle flap combined with a gluteal fasciocutaneous rotation flap and an acellular dermal matrix are appropriate for partial sacrectomy when local vasculature remains intact [65].

## 4. Results: Illustrative Case

### 4.1. Clinical Elements

**Figure 1:**
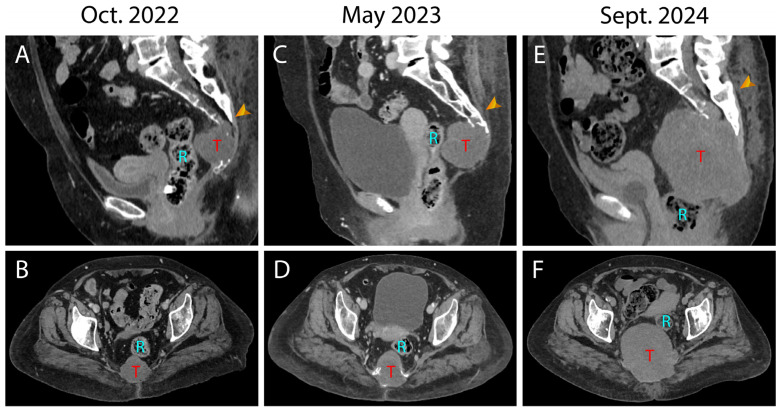
Preoperative CT imaging illustrating rapid tumor progression. (**A**,**B**), sagittal and axial contrasted CT scan image illustrating the tumor extension in 2022; (**C**,**D**), sagittal and axial contrasted CT scan image illustrating the tumor extension in 2023; (**E**,**F**), sagittal and axial contrasted CT scan image illustrating the tumor extension in 2024. Notice the significant progression in size of the tumor. Notice the well-defined adipose tissue plane between the rectum (blue capital letter R) and the tumor (red capital letter T) present in 2022 and 2023 but absent in 2024. Notice also the cranial progression of the tumor in the spinal canal from S4 in 2022 to S2–S3 in 2024 (orange arrowhead). The tumor is indicated by the red capital letter T. The rectum is indicated by the red capital letter T. Tumor progression in the spinal canal indicated by the orange arrowhead.

We present the case of a 66-year-old female, with a medical history of 45 pack-years of smoking, alcohol use, chronic obstructive pulmonary disease, and schizophrenia. The patient has been under radiological surveillance for a sacral lesion since 2022, initially investigated due to complaints of sacral and anal pain and unexplained lower limb edema. This initial CT revealed a 52 × 50 × 53 mm^3^ hypodense heterogeneous lesion centered on the lower sacral vertebrae, with associated lytic changes and bone infiltration (Figure 1A,B). At that time, the patient had a Karnofsky Performance Status (KPS) of 90. A follow-up CT in May 2023 showed a slight increase in the lesion’s size, prompting the tumor board to recommend an MRI for further characterization, considering the differential diagnosis of sarcoma versus chordoma (Figure 1C,D).

**Figure 2:**
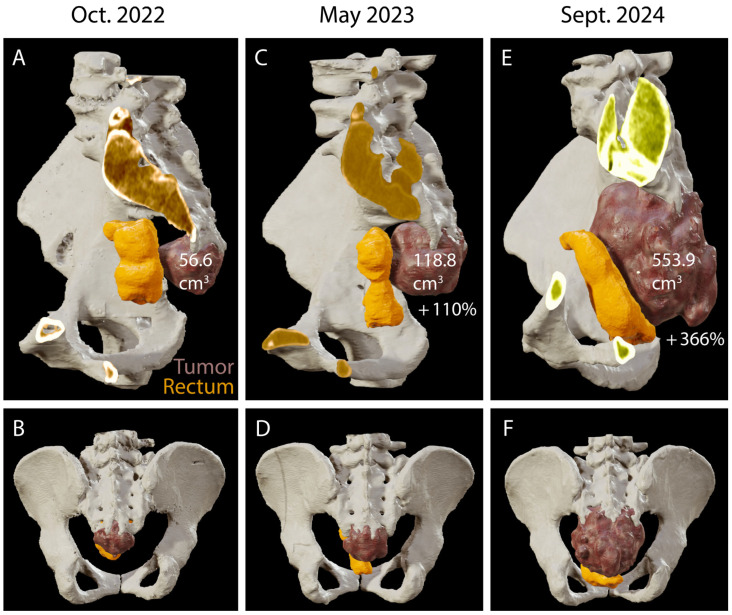
Notably, 3D reconstruction based on the preoperative CT and MR imaging illustrating tumor progression. (**A**–**F**), sagittal and coronal view of 3D reconstruction of the CT scans from Figure 1 illustrating the rapidly growing tumor and bony invasion in less than 2 years. Tumor volume is indicated in cm^3^ and growth since the last imaging in percentage. (**A**,**B**), sagittal and coronal view of 3D reconstruction of the CT scans from Figure 1. 2023; (**C**,**D**), sagittal and coronal view of 3D reconstruction of the CT scans from Figure 1 showing tumor progression (+110% since October 2022) with clear invasion of the 4th posterior sacral foramen; (**E**,**F**), sagittal and coronal view of 3D reconstruction of the CT scans from Figure 1. 2024 showing significant tumor progression with invasion of the 3rd posterior sacral foramen (+366% since May 2023). Notice how the tumor pushes the rectum ventrally. For the segmentation, automatic thresholding ROI was first generated and then manually corrected. The tumor is colored in brown and the rectum is colored in orange.

In June 2024, after initial reluctance, likely related to her psychiatric comorbidities, the patient consented to a needle biopsy, which confirmed the diagnosis of a sacral chordoma. A multidisciplinary team recommended en-bloc surgical resection of the tumor. In July 2024, the patient subsequently presented to the emergency department with acute, severe sacral pain and urinary difficulties. MR and CT imaging under general anesthesia showed a significant increase in tumor size (Figure 1E,F). Presacral and cranial extension was significant and the infiltration of the posterior sacral foramina reached up to S3 (Figure 2A–F).

**Figure 3:**
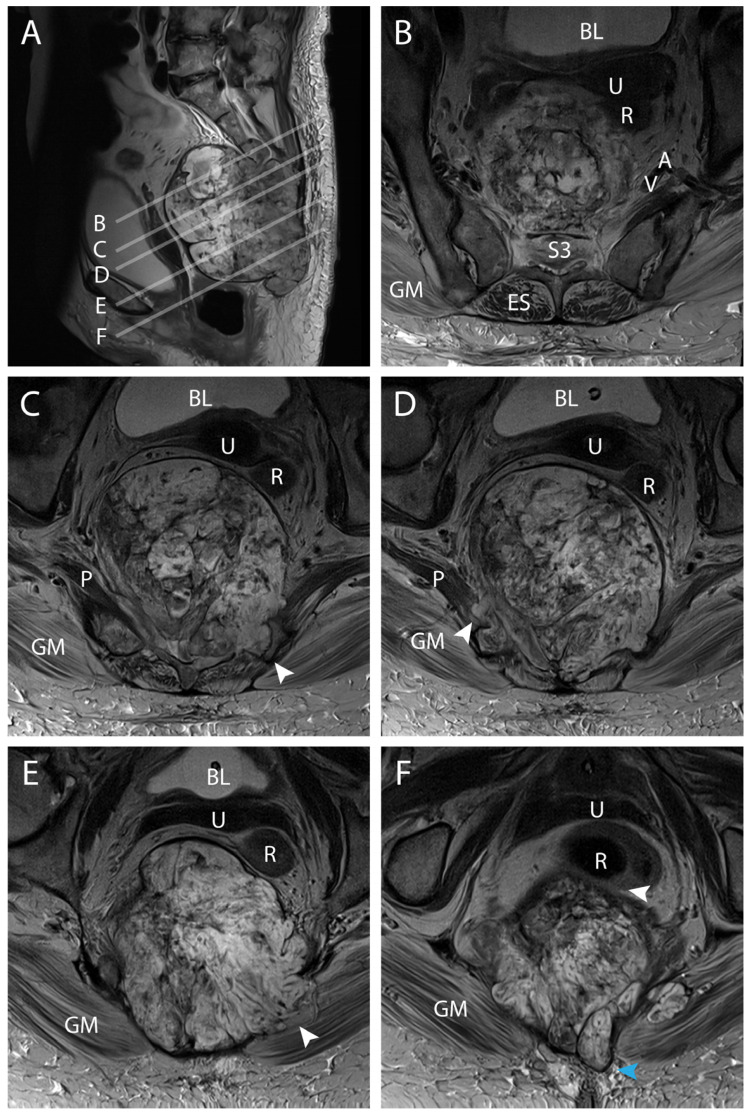
Preoperative MR scan illustrating local invasiveness. (**A**), midsagittal T2 MRI illustrating the tumor localization and the corresponding axial levels for (**B**–**F**); (**B**), axial plan from A showing the tumor invasion of the S3 soma and of the spinal canal; (**C**), axial plan from A showing the tumor invasion of the sacral ala on the left side at the level of S4 (white arrowhead); (**D**), axial plan from A showing the tumor invasion of the piriformis muscle on the right side at the level of S5 (white arrowhead); (**E**), axial plan from A showing the tumor invasion of the gluteus maximus on the left side under the level of the sacrum (white arrowhead); (**F**), axial plan from A showing the immediate contact of the tumor with the rectum in the lower pelvis (white arrowhead) and the tumor extension to the superficial tissue layers (blue arrowhead). BL = bladder; U = uterus; R = rectum; V + A = internal iliac vein and artery; GM = Gluteus maximus; P = Piriformis.

The tumor increased in size more than four times since May 2023 (Figure 1E,F and Figure 2). The MR scan showed extended invasion of the surrounding bony and soft tissue including infiltration of the spinal canal at the level S2–S3 close proximity to the internal iliac vessels (Figure 3A,B), bony invasion of the left sacral ala at the level S3 (Figure 3A,C), infiltration of the ischiococcygeus muscle on the right and gluteus maximus on the left (Figure 3A,D,E), immediate contact with the rectum and an extension to the superficial subcutaneous tissue (Figure 3A,F). En-bloc resection was scheduled. At this time, the patient had a Karnofsky Performance Status (KPS) of 70.

### 4.2. Preoperative Planning

**Figure 4:**
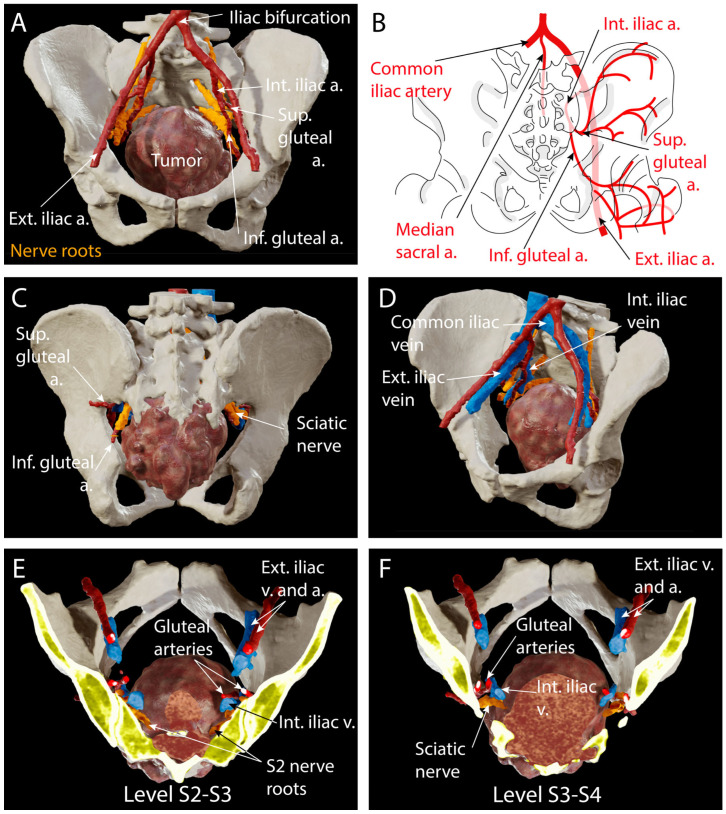
Notably, 3D reconstruction from preoperative contrast-enhanced CT scan for preoperative planning and assessment of vascular anatomy. (**A**) Notably, 3D reconstruction of the pelvis, sacrum, and last lumbar vertebra, the tumor, vascular anatomy, and L5, S1, and S2 nerve roots. This anterior view illustrates the anatomical relationship of the sacral ventral roots and the main arterial anatomy in close relation with the dorsal aspect of the tumor; (**B**), schematic of the arterial anatomy around the greater sciatic foramen; (**C**), dorsal view of the 3D reconstruction, showing the exiting sciatic nerve and gluteal arteries through the greater sciatic foramen; (**D**), antero-lateral view of the peritumoral pelvic vascular anatomy; (**E**), cranial view of an axial cut at S2–S3 junctional level, illustrating the tumor invasion of the spinal canal and the position of the L5–S1–S2 ventral nerve roots and arterio-venous anatomy. Notice the close proximity of the S2 nerve roots that were planned to be sacrificed during surgery; (**F**), cranial view of an axial cut at S3–S4 level, illustrating the tumor invasion of the sacrum and the position of the sciatic nerve and vessels. Tumor is colored in brown. Lombo-sacral nerves (L5–S2) are colored in orange. Arteries are colored in red. Veins are colored blue.

Preoperatively, MR and CT scans were ordered for surgical planning and intraoperative navigation. The pelvis and lombo-sacral bone were reconstructed and analyzed in 3D to prepare the level of the sacrectomy. In this case, bone invasion extended to the lower part of S2 and complete infiltration of the posterior sacral foramina of S3 (Figure 2E,F). Therefore, the bone cut was planned at level S1–S2 with preservation of the sacroiliac joint to avoid any need for stabilization. The L5 and S1–S2 nerve roots were reconstructed in 3D (Figure 4A–F) to assess anatomical variations and plan the dissection during tumor resection in order to avoid injury to the sciatic nerve as it exits the presacral space through the greater sciatic foramen ventrally to the piriformis muscle (Figure 4A,C).

Ligation of the dural sac was also planned caudal to the exiting roots S1 because of the close proximity of the S2 nerve roots to the tumor (Figure 4A,E,F). From an abdomino-pelvic point of view, resection of the rectum was decided upon as it was in direct contact with the tumor (Figure 1E,F, Figure 2E,F and Figure 3F) and ligation of the internal iliac vessels was planned due to their close proximity to the tumor (Figure 3B and Figure 4A–F). Sacrificing the internal iliac arteries is important for bleeding control and is allowed by the high number of anastomoses taking up the supply to the pelvic organs and internal part of the thigh. Subcutaneous tissue extension was assessed and a split skin incision was planned for a tailored extra-marginal skin cut (Figure 3F).

### 4.3. Surgical Technique

The surgical procedure was carried out in two distinct stages. The first stage of the procedure involved a laparoscopic abdominoperineal resection with the creation of a permanent left-sided colostomy, performed by the visceral surgery team. In the gynecological position and using an open laparoscopy technique, an adhesiolysis was first performed to mobilize the left colon, ensuring preservation of the left colic artery and ureter. A complete ureterolysis was carried out to prevent injury during the second stage of the surgery. The inferior mesenteric artery was transected using Ligasure. The colon was then divided, followed by a partial resection of the mesorectum, performed according to the Heald technique [108], extending to the S1 level. The internal iliac arteries, veins, and mediosacral vessels were ligated by placing Hem-o-lok clips proximally and distally on the arteries, following their bifurcation, and on the veins on both sides. In preparation for the posterior approach, a 10 × 10 cm gauze pad was placed on the specimen after securing the internal iliac vessels on both sides dorsally to the uterus and in the area where the rectum was resected. The anterior side of the rectum was completely freed from the vagina and an intersphincteric abdominal resection was performed, leaving the posterior side of the rectum and the mesorectum intact with the sacrum. An indocyanine green angiography indicated the need to resect an additional 5 cm of the descending colon at the colostomy site. The left colon was exteriorized at the stoma site, and the abdominal wall was reinforced with IPST Dynamesh and secured using GLUBRAND adhesive under laparoscopic guidance. Once the wounds were sutured, the patient could be repositioned in the prone position.

The second stage of the procedure, performed with the patient in a prone position, was conducted simultaneously by the neurosurgery and visceral teams, using a navigated posterior lumbosacral approach. A C-arm fluoroscopy device, combined with a Brainlab navigation system, was used to precisely identify the planned superficial and deep tissue resection margins, and the bone cut and to protect the sciatic nerve. For this purpose, the navigation reference clamp was securely attached to the spinous process of L4 after fluoroscopic verification, and was carefully oriented cranially (toward the patient’s head) to avoid interfering with the surgical field or the trajectory of the osteotomes used during the sacrectomy. Importantly, the sacrum and ilium are anatomically rigidly connected to the pelvis, and their relative position remains unchanged from preoperative imaging to the intraoperative setting. This inherent anatomical stability ensures that a surface match at S1 remains reliable and accurate for navigation throughout the iliosacral region. The accuracy of the 3D reconstruction was further confirmed intraoperatively by identifying key anatomical landmarks, including the sacroiliac joint and the sacral foramina. These served as control points to validate the accuracy of the navigation system. Thus, in this context, repetitive surface matching along the sacrum was not required, and a single registration at the posterior elements of S1 was sufficient to ensure accurate navigation during the osteotomy.

**Figure 5:**
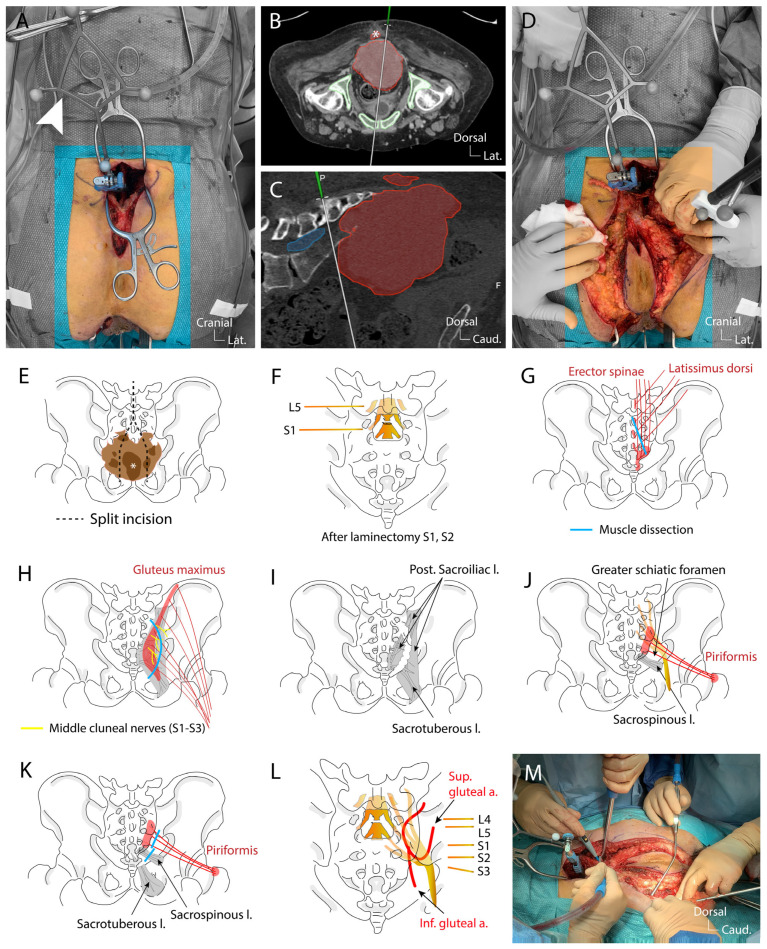
The surgical protocol of sacrectomy. (**A**), L4 spinous process is identified on lateral x-ray, a linear incision is made, the thoraco-lumbar fascia is incised and the paravertebral musculature is subperioestally dissected until complete exposure of the L4 and L5 spinous processes, autostatic retractors are positioned, a navigation star clamp is fixed to the L4 spinous process and a surface matching-based calibration is performed on the posterior elements of L5; (**B**), based on the matching between the preoperative CT and the matched calibrated navigation, a split incision (**E**) is performed in order to avoid the central cutaneous and subcutaneous tissues infiltrated by the tumor; (**C**), screenshot of the correlated preoperative CT scan during navigation to find the right level of dural sac ligature (tumor highlighted in red, dural sac in blue); (**D**), based on the navigation, guidance is obtained to accurately localize the pathological margins and perform the tailored S1–S2 laminectomy (**F**); (**E**), schematic of the navigation aided skin split incision; (**F**), schematic of the S1–S2 laminectomy and dural sac ligature just caudal to the S1 exiting roots; (**G**), schematic of the insertion site of the and latissimus dorsi on the sacrum detached during the first surgical steps; (**H**), schematic illustrating the sacroiliac insertion site of the gluteus maximus, the middle cluneal nerves are sacrificed during muscle detachment; (**I**), during the detachment of the gluteus maximus the posterior sacroiliac ligament and the sacrotuberous ligament needs to be cut close to the lateral border of the sacrum; (**J**), schematic showing after the cut made through the first layer of ligaments the deeper sitting sacrospinous ligament and piriform muscle covering the sciatic nerve as it exits through the greater sciatic foramen; (**K**), schematic illustrating the next step consisting of cutting through the sacrospinous ligament and detaching the piriform muscle both attached to the inner face of the sacrum to free the sacral piece; (**L**), schematic of a dorsal view of the sacrum after the last layers of muscles and ligaments attaching the sacrum to the iliac bone are cut revealing the sciatic nerve passing through the greater sciatic foramen surrounded by the gluteal vessels; (**M**), picture illustrating the eight hands working around the en-bloc resection just before the final osteotomy is performed.

The procedure began with an incision from L3 to the intergluteal fold, following lateral X-ray imaging. Subperiosteal dissection of the paravertebral muscles from L4 to S1 was performed, and the navigation clamp was secured onto the L4 spinous process (Figure 5A).

Surface matching was conducted using preoperative CT fused with MR imaging. The accuracy of the navigation was confirmed using exposed bony landmarks. The skin incision was then extended caudally in a split incision technique to include the superficially invaded soft tissue as identified by the navigation (Figure 5B,D,E). In parallel, the anus was closed with an Ethibond 2-0 suture, and an intersphincteric extralevator dissection was performed, ensuring healthy margins, with the right side dissected along the bone structure. Under the guidance of neuronavigation, the cranial extension of the tumor was identified (Figure 5C) and a laminectomy of S1–S2 was performed using a diamond burr to expose the dural sheath and the axillae of the S1 and S2 nerve roots on both sides (Figure 5F). The dural sheath was ligated with 3-0 Prolene sutures below the S1 nerve roots and transected between two ligatures to prevent cerebrospinal fluid leakage (Figure 5F). Regarding the use of augmented reality overlay through microscope integration, we acknowledge the usefulness of this technology in various spinal procedures. However, in our case, the dura at the sacral level is superficial and easily accessible due to the wide subperiosteal exposure. Exposure and ligature of the dura could easily be performed macroscopically; as such, a microscope was not necessary for this specific step. Furthermore, during osteotomy, the use of long osteotomes made microscope use impractical, as it would obstruct the surgical field. Instead, we relied on real-time 3D anatomical reconstructions displayed on the navigation screen to guide osteotomy trajectories and avoid tumor penetration. Next, the sacrospinalis muscles were transected (Figure 5G), and the gluteus maximus muscles were detached from the sacrum, sacrificing the branches of the middle cluneal nerves (S1–S3) innervating the buttocks (Figure 5H). The posterior sacroiliac and sacrotuberous ligaments were sectioned to free the lateral and inferolateral margins of the sacral ala and inferior sacrum (Figure 5I). This step reveals the sacrospinous ligament and piriformis muscles both attached to the ventral surface of the sacrum, which were also transected at their origins near the sacrum (Figure 5J,K). Beneath the piriformis muscle, the sciatic nerve (formed by the L4–S3 nerve roots) was identified and confirmed with navigation, and its origins from L5 and S1 were verified using direct stimulation with a monopolar neuromonitoring probe (Figure 5L). The superior and inferior gluteal vessels were identified, with the inferior vessels ligated (branches of the internal iliac artery exiting the presacral space via the greater sciatic foramen) (Figure 5L).

**Figure 6:**
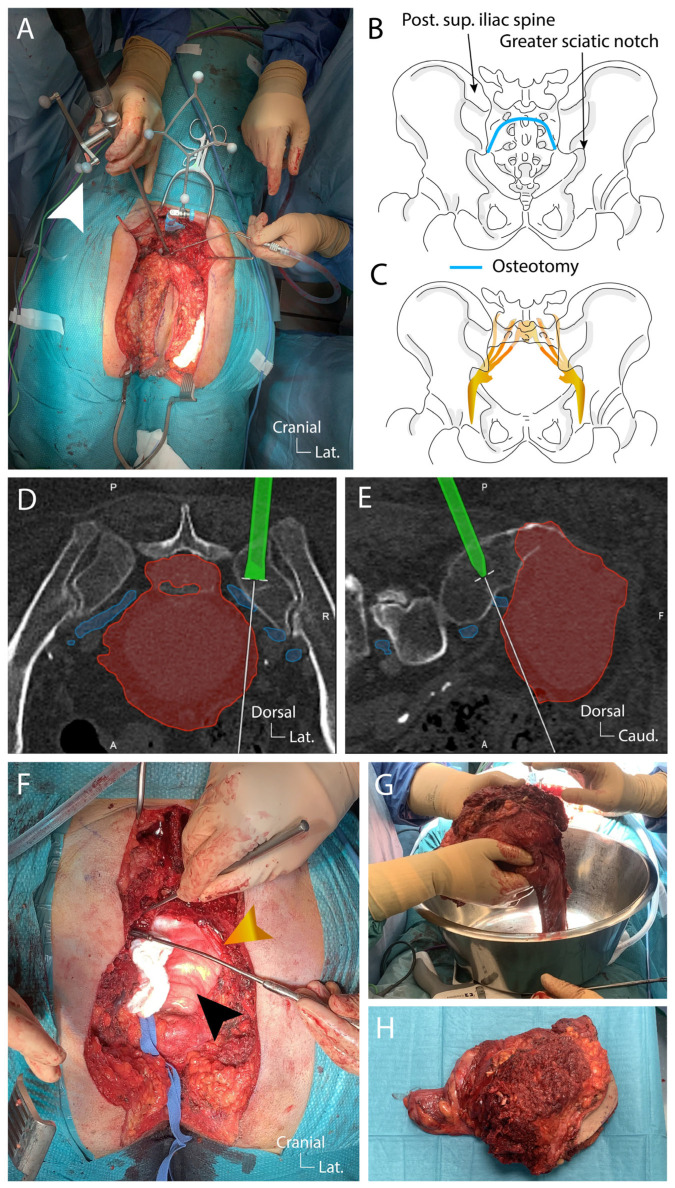
Second stage of the posterior approach: the navigated osteotomy and en-bloc removal of the pathological tissue. (**A**), dorsal view of the surgical field during the osteotomy (blue line) stage guided by navigation (the navigated osteotome is pointed by the white arrow head); (**B**), schematic of the osteotomy cutting between the S1 and S2 sacral vertebrae, extending laterally until the medial border of the postero-superior iliac spine and inferiorly to the greater sciatic notch; (**C**), schematic of the sacrectomy and the anatomical position of the sciatic nerve; (**D**,**E**), axial and sagittal intraoperative screenshots of the correlated CT/MR images and tumor/nerve roots reconstruction during navigation (tumor is highlighted in red, nerve roots in blue and osteotome in green; (**F**), dorsal view after en-bloc resection revealing the gauze left in place during the anterior approach in the pretumoral space (indicated by the black arrow head) and the sciatic nerve (indicated on the left side by the orange arrow head); (**G**,**H**), en-bloc pathological piece containing the rectum, tumor, sacrum, anus, piriformis muscles, and surrounding tissues including skin.

Once adequate exposure of the planned en-bloc piece was achieved and the sciatic nerve identified (Figure 5L), the sacrectomy was initiated as planned using navigated osteotomes (Figure 6A).

The resection began at the S1–S2 disc and extended laterally to the posterior superior iliac spine on the left side, reaching inferiorly to the greater sciatic notch (Figure 6B,C). The same steps were repeated on the right side. The navigation during this step is essential to preserve as much as possible the sacroiliac joint and to tailor the bone cut to preserve a safe margin (Figure 6D,E). During the osteotomy, continuous monitoring of the L5-S1 nerve roots was performed. After completing the osteotomy and securing the mediosacral vessels, the surgical team reached the 10 × 10 cm gauze placed during the ventral approach (Figure 6F). The specimen, consisting of the sacrum, tumor, rectum, anus, piriformis muscles, and surrounding tissues including skin, was removed en-bloc (Figure 6G,H). Throughout the procedure, the L5–S1 nerve roots were protected, with no changes in neuromonitoring signals. Hemostasis and irrigation were performed. A mesh was placed in the pelvic ring, secured inferiorly to the uterus and superiorly to the bone, with the Ovitex mesh protecting the sciatic nerves. Lumbopelvic stabilization was deemed unnecessary in this case. A muscle pad and Tachosil patch were placed over the durotomy site and a vacuum-assisted closure system (VAC) was set in the surgical cavity.

Six weeks later, surgical reconstruction of the sacral region defect was performed by using bilateral pediculated gracilis flaps, a Vicryl mesh and a double gluteal rotation flap.

### 4.4. Postoperative Course

**Figure 7:**
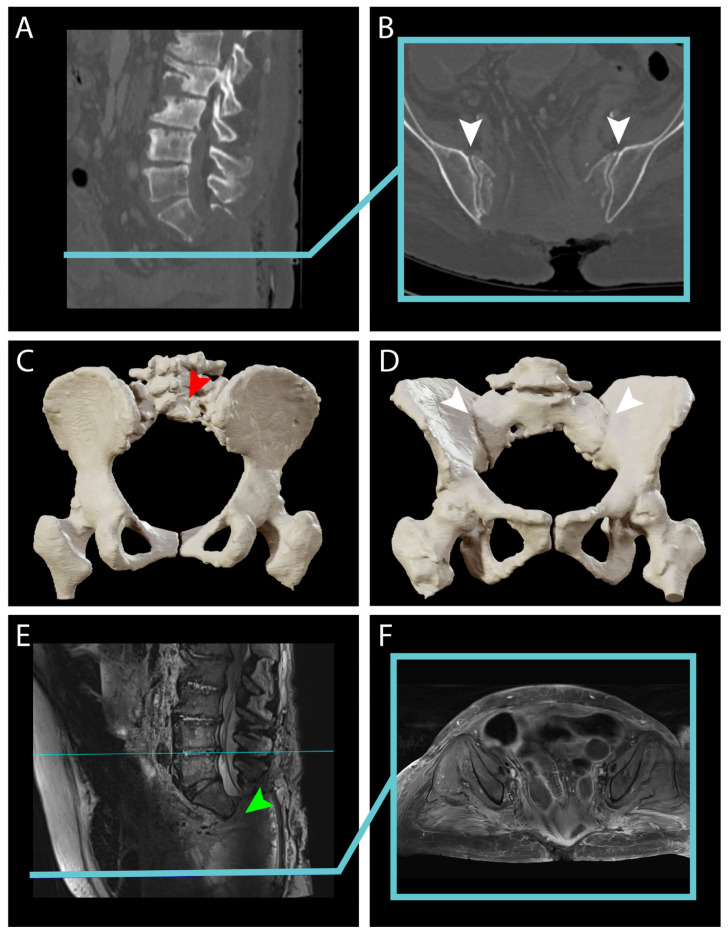
Postoperative CT and MRI scan. (**A**), sagittal image from CT scan showing the level of osteotomy at level S1–S2; (**B**) axial image from the same CT scan as A from the level indicated by the blue line showing the lateral limits of the osteotomy with preservation of the ilio-sacral joint (white arrows); (**C**), CT scan based 3D reconstruction illustrating the extension of the sacrectomy from a ventral view (red arrowhead indicates the extent of S1 laminectomy); (**D**), CT scan based 3D reconstruction illustrating the extension of the sacrectomy from a dorsal view with preservation of the sacroiliac joint (indicated by the white arrows); (**E**), sagittal image from MR scan showing the level of dural sac ligature (pointed by the green arrowhead); (**F**), axial image from MR scan at the level of the blue line in (**E**) illustrating the complete resection of the tumor with the dorsal herniation of the pelvic organs.

Postoperative CT and MRI scans confirmed successful en-bloc resection without immediate complications (Figure 7). The CT scan confirmed the preservation of the sacroiliac joint (Figure 7A–D). The MR scan confirmed the dural ligature at the level S1–S2 caudal to the emergence of the S1 nerve root (Figure 7E,F). The final pathology report identified a conventional chordoma with negative resection margins. However, on postoperative day 4, the patient experienced a perineal prolapse, which was managed with the implantation of a new mesh for pelvic floor reconstruction. On postoperative day 25, the patient developed septic shock, with blood cultures testing positive for *E. coli* and methicillin-resistant *Staphylococcus epidermidis*. She was started on Ceftriaxone and Metronidazole and taken to the operating room for removal and replacement of the vacuum-assisted closure (or VAC) system. Fortunately, the patient was able to be extubated promptly and transferred back to the surgical ward. On postoperative day 29, surgical reconstruction of the sacral region was performed using a bilateral pediculated gracilis flap, a gluteal rotation flap, and a Vicryl mesh, without any complications. Subsequently, on postoperative day 48, additional tissue debridement was required, and a new Prevena VAC system was placed. At this time, the patient had a Karnofsky Performance Status (KPS) of 50.

## 5. Discussion

The management of sacral chordomas represents a significant challenge due to their anatomical complexity, invasive growth patterns, and resistance to conventional therapies such as chemotherapy and radiation [27,28,29]. Achieving optimal oncological and functional outcomes requires a nuanced approach, balancing en-bloc resection with adequate surgical margins and the preservation of biomechanical stability and neurological function.

Numerous studies report that achieving negative resection margins is a key determinant of long-term survival and reduced recurrence in sacral chordomas [33,34,35,36,37,38]. En-bloc resection without tumor capsule violation remains the gold standard. However, the ability to achieve wide margins is often limited by the proximity of critical anatomical structures, particularly in tumors extending to the upper sacrum (S1 and S2) [48,50,55]. While postoperative radiation therapy has been associated with improved survival rates [5,7,42], its role remains adjunctive, underscoring the importance of surgical precision in achieving curative outcomes.

Given the complexity of sacral chordoma resection, multidisciplinary surgical teams are essential in optimizing patient outcomes, particularly for large or invasive tumors [26,38]. Neurosurgeons, colorectal surgeons, and plastic surgeons collaborate to address the dual challenges of achieving clear resection margins and minimizing perioperative risks [1,4,26,59,60]. Plastic surgeons play a critical role in reconstructing tissue defects, thereby reducing wound-related complications [62,64,65]. Despite these advancements, the added complexity of multidisciplinary interventions has not been definitively linked to better long-term outcomes and may increase perioperative risks such as longer surgical times and higher complication rates [1,26,38].

The choice of surgical approach is dictated primarily by tumor location and extent of invasion. Posterior-only approaches are favored for distal lesions below S3, where access to critical structures is not required [8,14]. This technique minimizes invasiveness and morbidity but is limited to smaller tumors without extensive pelvic or rectal involvement. Conversely, combined anterior-posterior approaches are essential for more complex tumors extending cephalad to S3 or involving the rectum and pelvic floor [1,14,48]. Although these approaches provide superior visualization and margin control, they are associated with higher complication rates and require advanced surgical planning using technologies like intraoperative navigation and 3D-printed anatomical models [1,24].

Sacral nerve preservation is a critical determinant of postoperative function, particularly for bladder, bowel, and sexual health. High sacrectomy (S1–S2) often results in significant neurological deficits, including motor impairment and sphincter dysfunction [38,74]. Preservation of at least one S2 root is associated with better continence outcomes, while sparing S3 roots offers additional benefits for bladder function [11,15,75]. These findings highlight the complex trade-offs between achieving oncological goals and preserving patient quality of life.

Sacrectomy, particularly at the S1–S2 level, compromises pelvic stability, necessitating robust reconstruction techniques. Resecting the sacroiliac joint by even one-third weakens the pelvic ring by 30%, and resection of tissue between S1 and S2 reduces stability by 50% [75,76]. Internal fixation techniques, such as double iliac screw fixation or the Galveston technique, are critical to restoring stability and enabling ambulation [12,13]. Regarding spinopelvic stabilization, extensive constructs are essential in cases of total sacrectomy, which results in complete spinopelvic dissociation. In situations where the osteotomy extends above the S1–S2 disc space or affects the sacroiliac articulation, additional stabilization using illio-sacral screws would be required to ensure construct stability [85,86,89]. In our case, the osteotomy was performed just below the S1–S1 disc space and did not compromise the sacroiliac joints. Therefore, primary iliosacral fixation was not deemed necessary.

The high rates of wound complications post-sacrectomy emphasize the importance of reconstructive strategies. Vertical rectus abdominis myocutaneous (VRAM) flaps are considered the gold standard for reconstructing large defects due to their reliability in obliterating dead space and reducing infection risk [63,99,100]. However, complications remain common, with up to 47% of patients requiring reoperation for wound issues [60]. While VRAM flaps are ideal for extensive defects and irradiated regions, local gluteal flaps may suffice for partial sacrectomy in patients with intact gluteal vasculature [65,99,104].

Emerging technologies, such as augmented reality and 3D-printed models, hold promise for improving surgical precision and outcomes, particularly in complex cases [1,24]. However, further research is needed to evaluate their impact on recurrence rates, functional outcomes, and long-term survival. Additionally, the role of multidisciplinary surgical teams warrants further investigation to determine whether their involvement translates to improved patient outcomes or only reflects the complexity of cases referred to specialized centers.

According to current guidelines for chordoma management, postoperative radiation—particularly proton beam therapy—is indeed essential [5,6,7,42]. In our patient’s case, proton beam therapy was planned and subsequently administered postoperatively.

## 6. Conclusions

Sacral chordoma management requires a delicate balance between oncological control, functional preservation, and biomechanical stability. Multidisciplinary collaboration and advanced surgical planning are critical, particularly for high sacral and invasive tumors. Advances in imaging and surgical techniques have reduced morbidity and improved precision, while careful preoperative planning and intraoperative navigation mitigate vascular and nervous risks associated with complex sacral anatomy. Despite this, high complication rates remain. Future research should focus on refining surgical techniques, integrating emerging technologies, and understanding the long-term outcomes of different reconstructive strategies to optimize patient care.

## Data Availability

Anonymized patient-related imaging data is available upon special request.

## Abbreviations

The following abbreviations are used in this manuscript:

PBRT	Proton beam radiation therapy
VRAM	Vertical rectus abdominis myocutaneous flap
VAC	Vacuum-assisted closure
